# The Cell Adhesion Molecule Necl-4/CADM4 Serves as a Novel Regulator for Contact Inhibition of Cell Movement and Proliferation

**DOI:** 10.1371/journal.pone.0124259

**Published:** 2015-04-20

**Authors:** Shota Yamana, Amina Tokiyama, Kiyohito Mizutani, Ken-ichi Hirata, Yoshimi Takai, Yoshiyuki Rikitake

**Affiliations:** 1 Division of Cardiovascular Medicine, Department of Internal Medicine, Kobe University Graduate School of Medicine, Kobe, Hyogo, Japan; 2 Division of Signal Transduction, Department of Biochemistry and Molecular Biology, Kobe University Graduate School of Medicine, Kobe, Hyogo, Japan; 3 Division of Molecular and Cellular Biology, Department of Biochemistry and Molecular Biology, Kobe University Graduate School of Medicine, Kobe, Hyogo, Japan; 4 Division of Pathogenetic Signaling, Department of Biochemistry and Molecular Biology, Kobe University Graduate School of Medicine, Kobe, Hyogo, Japan; Northwestern University, UNITED STATES

## Abstract

Contact inhibition of cell movement and proliferation is critical for proper organogenesis and tissue remodeling. We show here a novel regulatory mechanism for this contact inhibition using cultured vascular endothelial cells. When the cells were confluently cultured, Necl-4 was up-regulated and localized at cell–cell contact sites where it *cis*-interacted with the vascular endothelial growth factor (VEGF) receptor. This interaction inhibited the tyrosine-phosphorylation of the VEGF receptor through protein-tyrosine phosphatase, non-receptor type 13 (PTPN13), eventually reducing cell movement and proliferation. When the cells were sparsely cultured, Necl-4 was down-regulated but accumulated at leading edges where it inhibited the activation of Rho-associated protein kinase through PTPN13, eventually facilitating the VEGF-induced activation of Rac1 and enhancing cell movement. Necl-4 further facilitated the activation of extracellular signal-regulated kinase 1/2, eventually enhancing cell proliferation. Thus, Necl-4 serves as a novel regulator for contact inhibition of cell movement and proliferation cooperatively with the VEGF receptor and PTPN13.

## Introduction

Contact inhibition of cell movement and proliferation is essential for proper morphogenesis and maintenance of tissue architecture during embryonic development and regeneration in adults [1,2]. Deregulation of contact inhibition is thought to play a role in tumorigenesis. Contact inhibition is a phenomenon that can be observed between two moving and proliferating cells in culture. When the cells collide, they gradually reduce movement and proliferation, and eventually stop moving and proliferating [1,2]. To understand the molecular mechanisms underlying the contact inhibition, the molecular mechanisms for cell—cell adhesion, cell movement, cell proliferation, and mutual interactions among these three cellular responses have been investigated. Until about two decades ago, cadherins were the only known critical cell—cell adhesion molecule (CAM) for cell—cell adhesion, and growth factor receptors and integrins were shown to be involved in cell movement and proliferation by cooperatively regulating the intracellular signals, including the small G proteins Rho and Ras, respectively. Although cadherins, growth factor receptors, and integrins were known to contribute to contact inhibition, these molecules could not completely account for its mechanisms. Our understanding of contact inhibition has progressed since the discovery of novel pathways, including the Hippo, merlin, and Eph-ephrin signaling pathways. Cadherins, through catenins, most likely regulate merlin, a product of the tumor suppressor gene *merlin/Nf2*, and merlin subsequently activates the Hippo pathway, which inhibits cell proliferation by inactivating the transcriptional coactivator YAP [3–6]. Eph—ephrin *trans*-interactions initiate bidirectional signaling within the receptor- and ligand-expressing cells. Activation of the EphA receptor by *trans*-interacting with ephrin in neighbouring prostate cancer cells causes the retraction of their movement [7].

We have shown that another family of CAMs nectins, consisting of four members (nectin-1 to nectin-4), regulate cell—cell adhesion, cell movement, and cell proliferation in various types of cells [8,9]. In epithelial cells, the *trans*-interactions of nectins induce the initial cell—cell adhesion, and then recruit cadherins to the nectin-based adhesion sites, eventually establishing adherens junctions (AJs) [8,9]. During or after AJ formation, nectins initially recruit junctional adhesion molecules (JAMs) to the adhesion sites, followed by the recruitment of occludin and claudins to the apical side of AJs in cooperation with cadherins, establishing tight junctions (TJs) [8,9]. Thus, cell—cell adhesion progresses in multiple stages. We have identified nectin-like molecule-5 (Necl-5), a member of the Necl family of proteins consisting of five members (Necl-1 to Necl-5), as the third regulatory factor for cell movement and proliferation, in addition to growth factor receptors and integrins [10]. Necl-5 forms a complex with PDGF receptor and integrin α_V_β_3_, and is localized at the leading edges of migrating NIH3T3 cells in response to PDGF [10]. This complex regulates dynamic activation and inactivation of the small G proteins Rap1, Rac, and RhoA, thereby inducing the formation of leading edge structures, such as lamellipodia, peripheral ruffles, and focal complexes, required for cell migration [10]. This complex also enhances cell proliferation by inhibiting sprouty2, a negative regulator of the Ras—extracellular signal-regulated kinase (ERK) pathway [11]. When two cultured NIH3T3 cells collide, Necl-5 at the leading edges *trans*-interacts with nectin-3 on the surface of the adjacent cells [12]. Necl-5 is down-regulated from the cell surface by endocytosis, and the nectin-3 retained on the cell membrane *trans*-interacts with nectin-1, initiating cell—cell adhesion and subsequently recruiting cadherins to form AJs [13]. Thus, nectins and Necl-5 cooperatively regulate cell—cell adhesion, cell movement, and cell proliferation, and play a role at the initiation stage of contact inhibition of cell movement and proliferation. Although contact inhibition has not been shown to occur at multiple stages, accumulating evidence suggests that contact inhibition is divided into at least two stages: the initiation stage with a decrease in cell movement and proliferation; and the maintenance stage with establishment of cell—cell adhesion and a stationary phase.

We investigated here whether Necl-4 (also termed TSLL2, IGSF4C, SynCAM4, or CADM4) [14–16] also regulated the contact inhibition of cell movement and proliferation, because it binds protein-tyrosine phosphatase, non-receptor type 13 (PTPN13, also termed PTP-BL, PTP-BAS, PTP-L1, or FAP1), a putative tumor suppressor, through the cytoplasmic region of Necl-4, and inhibits the heregulin-induced activation of the ErbB2/ErbB3 signaling through PTPN13 and the phorbol ester-induced disassembly of hemidesmosomes [17]. Necl-4 acts as a tumor suppressor and its expression is lost or markedly reduced in various human cancer cell lines [14,16,18]. To investigate the role of Necl-4 in contact inhibition, we used cultured endothelial cells (ECs) as a model cell line for several reasons. First, ECs have frequently been used to study this phenomenon: under sparse conditions, ECs are highly sensitive to stimulation by growth factors, including VEGF [19], and their cellular status is comparable to moving and proliferating cells. When they reach confluence and establish firm cell—cell junctions, ECs lose the ability to respond to growth factors and switch to a quiescent condition [20,21]. Second, ECs can be easily analyzed for tubulogenesis, a form of organogenesis, *in vitro* [22]. Lastly, we have a substantial amount of information on nectins and Necls in ECs [8,9,23,24]. We show here that Necl-4 serves as a novel regulator for contact inhibition of cell movement and proliferation at the initiation stage.

## Materials and Methods

### Antibodies, plasmids, and reagents

Rabbit anti-Necl-1 polyclonal antibody (pAb), rat anti-Necl-2 mAb, and chicken antisera against Necl-5 mAb were prepared as described previously [25–27]. Rabbit anti-Necl-3 pAb was raised against the 2nd loop of mouse Necl-3 (aa 155–216). Rabbit anti-Necl-4 pAb was raised against the cytoplasmic tail of mouse Necl-4 (aa 345–388). Alexa 488-conjugated isolectin B4 (Life Technologies, Carlsbad, CA, USA), goat anti-vascular endothelial cadherin (VE-cadherin) pAb (sc-6458, Santa Cruz Biotechnology), mouse anti-Necl-4/SynCAM4 mAb (UC Davis/NIH NeuroMab Facility, Davis, CA, USA), mouse anti-human Necl-5/CD155 mAb (MAB2530, R&D Systems, Inc., Minneapolis, MN), rabbit anti-nectin-2 mAb (ab135246, Abcam, Cambridge, UK), goat anti-nectin-3 pAb (sc-14806, Santa Cruz Biotechnology, Santa Cruz, CA), mouse anti-vinculin mAb (V4505, Sigma-Aldrich, St. Louis, MO, USA), Alexa 488-conjugated phalloidin (Life Technologies), mouse anti-afadin/AF6 mAb (610732, BD Biosciences, San Jose, CA, USA), rabbit anti-Rap1 pAb (sc-65, Santa Cruz Biotechnology), mouse anti-FLAG mAb (F1804, Sigma-Aldrich), rabbit anti-FLAG pAb (F7425, Sigma-Aldrich), rabbit anti-VEGF receptor (VEGFR) 1 pAb (sc-316, Santa Cruz Biotechnology), rabbit anti-phospho-VEGFR2 (Y1175) pAb (#3770, Cell Signaling Technology, Danvers, MA, USA), rabbit anti-VEGFR2 pAb (sc-504, Santa Cruz Biotechnology), rabbit anti-p44/42 MAPK pAb (#9102, Cell Signaling Technology), rabbit anti-phospho-p44/42 MAPK (T202/Y204) pAb (#9101, Cell Signaling Technology), rabbit anti-phospho-myosin phosphatase target subunit 1 (MYPT1)/myosin-binding subunit (MBS) (Thr853) pAb (#4563, Cell Signaling Technology), rabbit anti-MYPT1/MBS pAb (#2634, Cell Signaling Technology), mouse anti-Rac1 mAb (610650, BD Biosciences), rabbit anti-PTPN13 pAb (PAB0256, Abnova, Taipei, Taiwan), and mouse anti-actin mAb (sc-8432, Santa Cruz Biotechnology; MAB1501, Merck Millipore, Billerica, MA, USA) were purchased from the indicated suppliers. Fluorophore (FITC and Cy3)-conjugated secondary antibodies were purchased from Jackson ImmunoResearch Laboratories (West Grove, PA, USA) and Merck Millipore. HRP-conjugated secondary antibodies were purchased from GE Healthcare Bioscience (Pittsburgh, PA, USA). 4′,6-Diamidino-2-phenylindole dihydrochloride (DAPI) was purchased from Nacalai Tesque, Inc. (Kyoto, Japan). pCAGIPuro-FLAG-Necl-4, pFLAG-CMV1-Necl-4-ΔCP, and pFLAG-CMV1-Necl-4-ΔEC were prepared as described.[17] pCI-neo-VEGFR1 and pCI-neo-VEGFR2 were prepared as described [23]. Human recombinant VEGF was purchased from Wako Pure Chemical Industries, Ltd. (Osaka, Japan). Growth factor-reduced Matrigel matrix without phenol red was purchased from BD Biosciences. Y-27632 and fasudil were purchased from Merck Millipore.

### Cell culture and transfection experiment

Primary cultures of human umbilical vein ECs (HUVECs) were obtained from Lonza (Basel, Switzerland) and maintained at 37°C using Endothelial Cell Growth Medium 2 (Lonza and PromoCell, Heidelberg, Germany) as described previously [23]. Cells between passages 3 and 8 were used for each experiment. Experiments were performed with sparse (25% confluency) and confluent (100% confluency) cell cultures, in collagen-coated 60- or 100-mm dishes. To obtain 100% confluency, the cells were seeded at a density of 1×10^6^ cells per 60-mm dish or 3×10^6^ cells per 100-mm dish in Endothelial Basal Medium-2 (EBM-2, Lonza) and cultured for 24 h. To obtain sparse (25%) confluency, the cells were seeded at a density of 2.5×10^5^ cells per 100-mm dish in EBM-2 and cultured for 24 h. For siRNA experiments, HUVECs were transfected with Stealth RNAis for Necl-4, PTPN13, Rap1, afadin or non-silencing negative control (Life Technologies) using Lipofectamine RNAiMAX (Life Technologies) according to the manufacturer’s instructions. Forty-eight h after transfection, cells were used for experiments. For transfection of plasmids into HUVECs, an electroporation method with Amaxa HUVEC Nucleofector Kit (Lonza) was used according to the manufacturer’s instructions. HEK293 cells were cultured and transfected with plasmids using Lipofectamine 2000 (Life Technologies) according to the manufacturer’s instructions as described previously [23].

### Wound-healing assays

Wound-healing assays were performed as described previously [24]. In brief, confluent HUVECs on 24-well plates coated with 10 μg/ml type I collagen or 5 μg/ml vitronectin were serum-starved in EBM-2 plus 0.1% FBS for 4 h. The monolayer was scratched with a sterile 10-μl pipette tip. The cells were gently washed with warm EBM-2 medium to remove detached cells and then cultured for 20 h in EBM-2 plus 2% FBS in the presence or absence of 50 ng/ml of VEGF.

### Cell proliferation

HUVECs on 24-well (5×10^3^ cells per well) or 96-well (5×10^3^ cells per well) plates coated with type I collagen were serum-starved in EBM-2 plus 0.1% FBS at 37°C in 5% CO_2_. After 4 h, the medium was replaced with EBM-2 plus 2% FBS in the presence or absence of 50 ng/ml VEGF at 37°C in 5% CO_2_. At the indicated time points, the numbers of cells on 96-well plates were quantified by crystal violet staining or HUVECs cultured on 24-well plates were detached and the number of the cells was counted.

### Capillary-like network formation (Tubulogenesis assay)

Capillary-like network formation on Matrigel was performed as described previously [24]. In brief, 24-well culture plates were coated with 300 μl of growth factor-reduced Matrigel per well and HUVECs, which had been serum-starved in EBM-2 plus 0.1% FBS for 4 h, seeded on coated plates at a density of 1×10^5^ cells per well in EBM-2 plus 1% FBS in the presence or absence of 50 ng/ml VEGF, followed by incubation for 20 h at 37°C. Total tube length was measured using ImageJ software (NIH, Bethesda, MD, USA).

### Immunofluorescence microscopy

Immunofluorescence microscopy was performed as described previously [24]. In brief, cryostat sections of C57/BL6 mouse tissues were fixed with acetone/methanol (1:1), incubated with HistoVT one (Nacalai Tesque Inc.) for 20 min at 56°C, and then incubated with 1% BSA, 10% normal goat serum, and 0.5% Triton X-100 in PBS for 30 min at room temperature. HUVECs were fixed with acetone/methanol (1:1) or 4% paraformaldehyde in PBS for 15 min and 0.2% Triton X-100 in PBS for 10 min at room temperature and incubated with 3% BSA in PBS overnight at 4°C. The samples were stained with the indicated antibodies (1:100), and then with appropriate fluorophore-conjugated secondary antibodies (1:200). Confocal image analyses were performed on a confocal laser-scanning microscope (LSM700, Carl Zeiss) using 20× or 63× objective lenses (Plan Apochromat with a numerical aperture of 0.8 DICIII and Plan Apochromat with a numerical aperture of 1.4 oil DICIII, Carl Zeiss). Images captured on the LSM 700 at room temperature were analyzed using ZEN acquisition software (Carl Zeiss).

### Western blotting, co-immunoprecipitation assays, and pull-down assays

Western blotting and co-immunoprecipitation assays were performed as described previously [24]. The primary and secondary antibodies were used at 1:1000 and 1:2000, respectively. ROCK activity was measured by MYPT1/MBS (T853) phosphorylation as described previously.[28] Pull-down assays were performed using GST-fused with p21-activated kinase (PAK)-Cdc42/Rac-interactive binding (CRIB) as described previously [29]. The signals were detected using the Amersham Imager 600 (GE Healthcare Bioscience). Densitometric analysis was performed using ImageJ software.

### Quantitative real-time reverse transcriptase PCR (qPCR)

qPCR was performed as described previously [23]. Total RNA was isolated from HUVECs using TRIzol Reagent (Invitrogen). A ReverTra Ace qPCR RT Kit (Toyobo) was used for RT. Synthesised cDNAs were subjected to real-time PCR using a 7500 Real-Time PCR System (Applied Biosystems) with a SYBR Premix Ex Taq II (Takara Bio). The following primers were used: Necl-4 (forward, 5′-GAGGGCGCTCTACGTACTTG-3′; reverse, 5′-TTGCTTCTCCCTGTTCATCC-3′), and GAPDH (forward, 5′-TGTGTCCGTCGTGGATCTGA-3′; reverse, 5′-TTGCTGTTGAAGTCGCAGGAG-3′). GAPDH was used for normalisation, and the comparative threshold method was used to assess the relative abundance of the targets.

### Statistical analysis

All experiments were conducted at least three times, and the results are expressed as the means ± SD. Statistical comparisons were performed using a one-way ANOVA with a Tukey—Kramer post-hoc test or a two-way ANOVA with a Bonferroni post-hoc test, as appropriate, where *P*<0.05 was considered statistically significant (GraphPad Prism version 5.0; GraphPad Software Inc., La Jolla, CA, USA).

## Results

### Necl-4 is localized at cell—cell contact sites and leading edges depending on cell density

**Fig 1:**
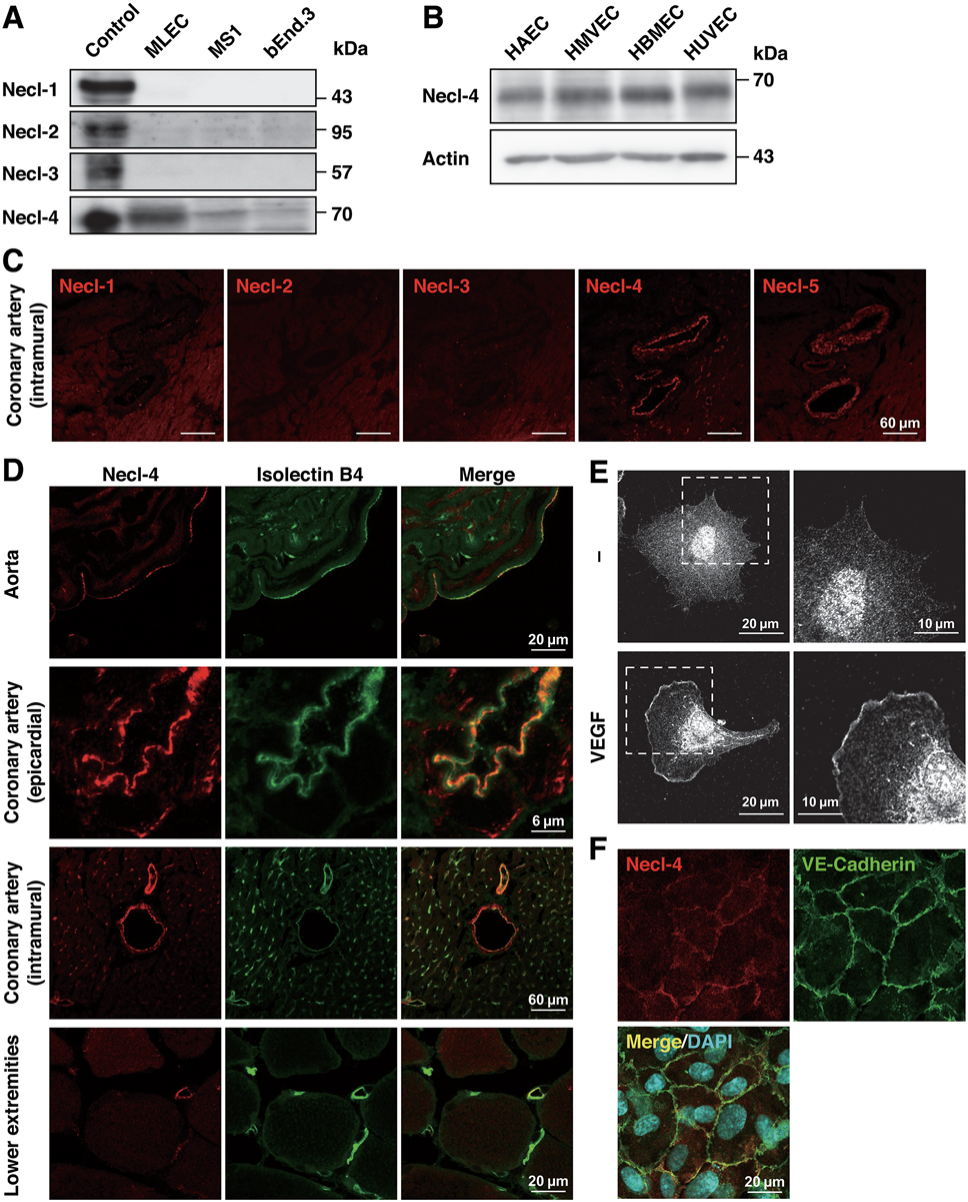
Necl-4 is localized at cell—cell contact sites and leading edges of ECs depending on cell density. **A**, Expression of Necls in cultured mouse ECs. Cell lysates were subjected to Western blotting using anti-Necl-4 mAb. MLEC, primary-cultured mouse lung EC; MS1, mouse pancreatic islet EC line; bEnd.3, mouse brain EC line. HEK293 cells transfected with each Necl-expressing plasmid were used as controls. **B**, Expression of Necl-4 in cultured human ECs. Cell lysates were subjected to Western blotting using the indicated antibodies. HAEC, human aortic EC; HMVEC, human lung microvascular EC; HBMEC, human brain microvascular EC; HUVEC, human umbilical vein EC. **C**, Expression of Necls in mouse coronary artery. Sections were stained with the antibodies against Necls. **D**, Expression of Necl-4 in mouse blood vessels. Sections were stained with anti-Necl-4 pAb and isolectin B4. **E**, Localization of Necl-4 at the leading edge. HUVECs were cultured in the presence or absence of 50 ng/ml VEGF and stained with anti-Necl-4 mAb. Representative low (left lane) and high (right lane) magnification images are shown (*n* = 3). **F**, Localization of Necl-4 confluent conditions. HUVECs were cultured until they formed cell—cell contact and double-stained with anti-Necl-4 mAb and anti-VE-cadherin pAb. Representative images are shown (*n* = 3).

We first analyzed the expression in cultured ECs of Necls other than Necl-5 for which expression was previously reported [23,30]. Necl-4, but not Necl-1, Necl-2 or Necl-3, was expressed to various degrees in cultured mouse ECs (Fig 1A). Cross-reaction was not seen among the antibodies used (S1 Fig). Necl-4 was also expressed in various cultured human ECs (Fig 1B). The immunofluorescence signals for Necl-4 and Necl-5, but not for Necl-1, Necl-2 or Necl-3, were observed in mouse intramural coronary arteries (Fig 1C). The signal for Necl-4 was co-localized with that for the endothelial marker isolectin B4 in various vessels, suggesting Necl-4 expression in ECs (Fig 1D). Thus, in addition to Necl-5 [23,30], Necl-4 is also expressed in ECs *in vitro* and *in vivo*.

We then analyzed the localization of Necl-4 in cultured HUVECs. Under sparse conditions, the signal for Necl-4 was observed at the leading edge of a single VEGF-stimulated HUVEC (Fig 1E). Under confluent conditions, the signal for Necl-4 was concentrated at the cell—cell contact sites, where it was co-localized with that for VE-cadherin (Fig 1F), suggesting that Necl-4 acts as a CAM [16]. These results show a cell density-dependent differential localization of Necl-4.

### Expression of Necl-4 is regulated by cell density through Rap1 and afadin

**Fig 2:**
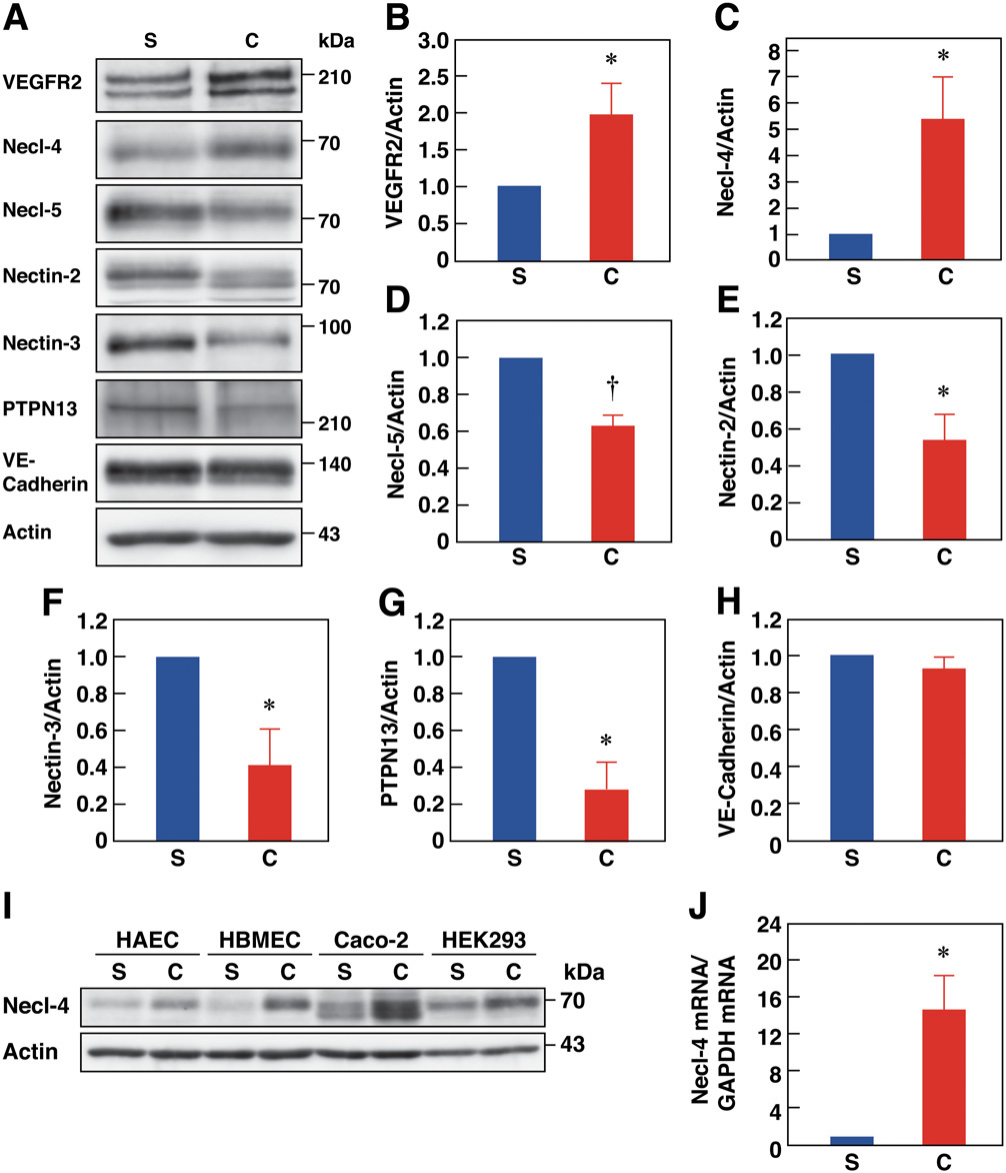
Expression of Necl-4 is regulated by cell-density through Rap1 and afadin in ECs. **A–H**, Comparison of the expression levels under sparse vs. confluent conditions. Lysates of HUVECs cultured under sparse (S, 25% confluence) or confluent (C, 100% confluence) conditions were subjected to Western blotting using the indicated antibodies (*n* = 4). *P<0.05; †*P*<0.01 vs. 25% confluence. **I**, Up-regulation of Necl-4 protein by confluence. Lysates of HAECs, HBMECs, the human colon epithelial cancer cell line Caco-2, and HEK293 cells, which were cultured under sparse or confluent conditions and subjected to Western blotting using the indicated antibodies (*n* = 3). **J**, Up-regulation of Necl-4 mRNA expression by confluence. RNAs extracted from HUVECs cultured under sparse (S, 25% confluence) or confluent (C, 100% confluence) conditions were subjected to qPCR (*n* = 4). *P<0.05 vs. 25% confluence.

To examine whether expression levels and functional roles of Necl-4 differ between sparse and confluent conditions, we first compared the expression levels of Necl-4 under these different conditions. HUVECs exhibited a confluence-dependent up-regulation of Necl-4 (Fig 2A and 2C). This was also observed in other EC types and in epithelial cells (Fig 2I). When HUVECs reached confluence, Necl-5, nectin-2, nectin-3, and PTPN13 were down-regulated, VEGFR2 was up-regulated, and VE-cadherin levels did not change (Fig 2A, 2B and 2D–2H). Confluence-dependent up-regulation of Necl-4 mRNA was observed (Fig 2J). Cell—cell adhesion was disturbed in Rap1- or afadin-knockdown HUVECs [24], and the confluence-dependent up-regulation of Necl-4 protein and mRNA was inhibited in Rap1- or afadin-knockdown cells (S2 Fig). Thus, Necl-4 expression is suggested to be regulated through Rap1 and afadin in a cell density-dependent manner.

### Necl-4 interacts with VEGFR2 and inhibits its activation, signaling, and cellular responses in confluently cultured ECs

**Fig 3:**
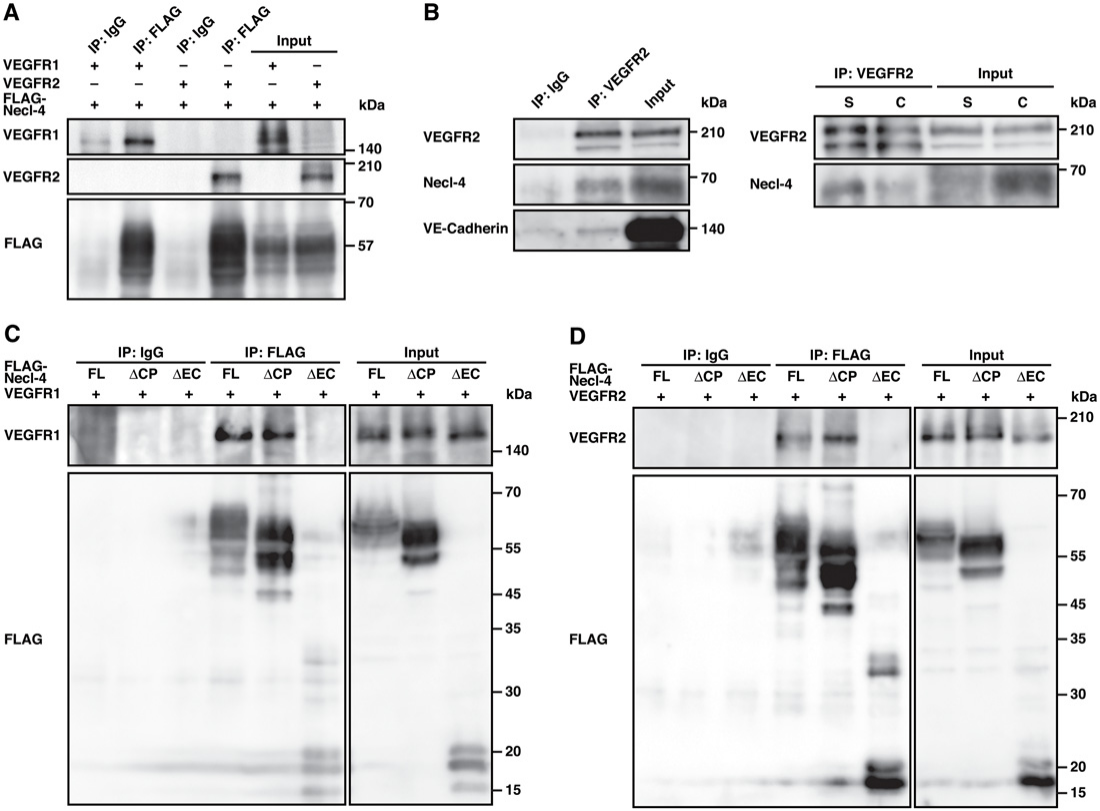
Necl-4 interacts with VEGFR1 and VEGFR2 through their extracellular regions. **A**, Interaction of Necl-4 with VEGFR1 and VEGFR2. HEK293 cells were transfected with FLAG-tagged Necl-4 and either VEGFR1 or VEGFR2. Cell lysates were subjected to co-immunoprecipitation assay using IgG as a control or the anti-FLAG mAb and samples were assessed by Western blotting using the indicated antibodies. **B**, Interaction of endogenous Necl-4 with endogenous VEGFR2 in ECs. Lysates of HUVECs cultured under sparse (S, 25% confluence) or confluent (C, 100% confluence) conditions were subjected to co-immunoprecipitation assays using IgG as a control or the anti-VEGFR2 pAb and samples were assessed by Western blotting using the indicated antibodies. **C and D**, Interaction of extracellular region of Necl-4 with VEGFR1 and VEGFR2. HEK293 cells were transfected with VEGFR1 (**C**) or VEGFR2 (**D**) and FLAG-tagged Necl-4, Necl-4-ΔCP, or Necl-4-ΔEC. Cell lysates were subjected to co-immunoprecipitation assay using IgG as a control or the anti-FLAG mAb. Samples were assessed by Western blotting using the indicated antibodies.

Of the two VEGF receptors, VEGFR1 and VEGFR2, the responses to VEGF for movement and proliferation are mainly mediated by VEGFR2 [31,32]. Because Necl-4 was localized at cell—cell contact sites in confluently cultured ECs, we hypothesised that Necl-4 might interact with VEGFR2 and coordinate its activation, signaling, and cellular responses under confluent conditions. We therefore first examined whether Necl-4 could interact with VEGFR1 and VEGFR2. In human embryonic kidney (HEK) 293 cells where FLAG-Necl-4 was co-expressed with VEGFR1 or VEGFR2, both VEGFR1 and VEGFR2 were co-immunoprecipitated with FLAG-Necl-4 (Fig 3A). Consistent with this, endogenous Necl-4 was co-immunoprecipitated with endogenous VEGFR2 in ECs, as VE-cadherin was co-immunoprecipitated (Fig 3B). Compared with sparse conditions, the amount of co-immunoprecipitated Necl-4 was decreased under confluent conditions (Fig 3B), although the amount of PTPN13 co-immunoprecipitated with VEGFR2 was increased (data not shown). In HEK293 cells where various Necl-4 mutants were co-expressed with VEGFR1 or VEGFR2, both VEGFR1 and VEGFR2 were co-immunoprecipitated with FLAG-Necl-4 and FLAG-Necl-4-ΔCP, but hardly with FLAG-Necl-4-ΔEC (Fig 3C and 3D). These results indicate that Necl-4 interacts with VEGFR2 through the extracellular region.

**Fig 4:**
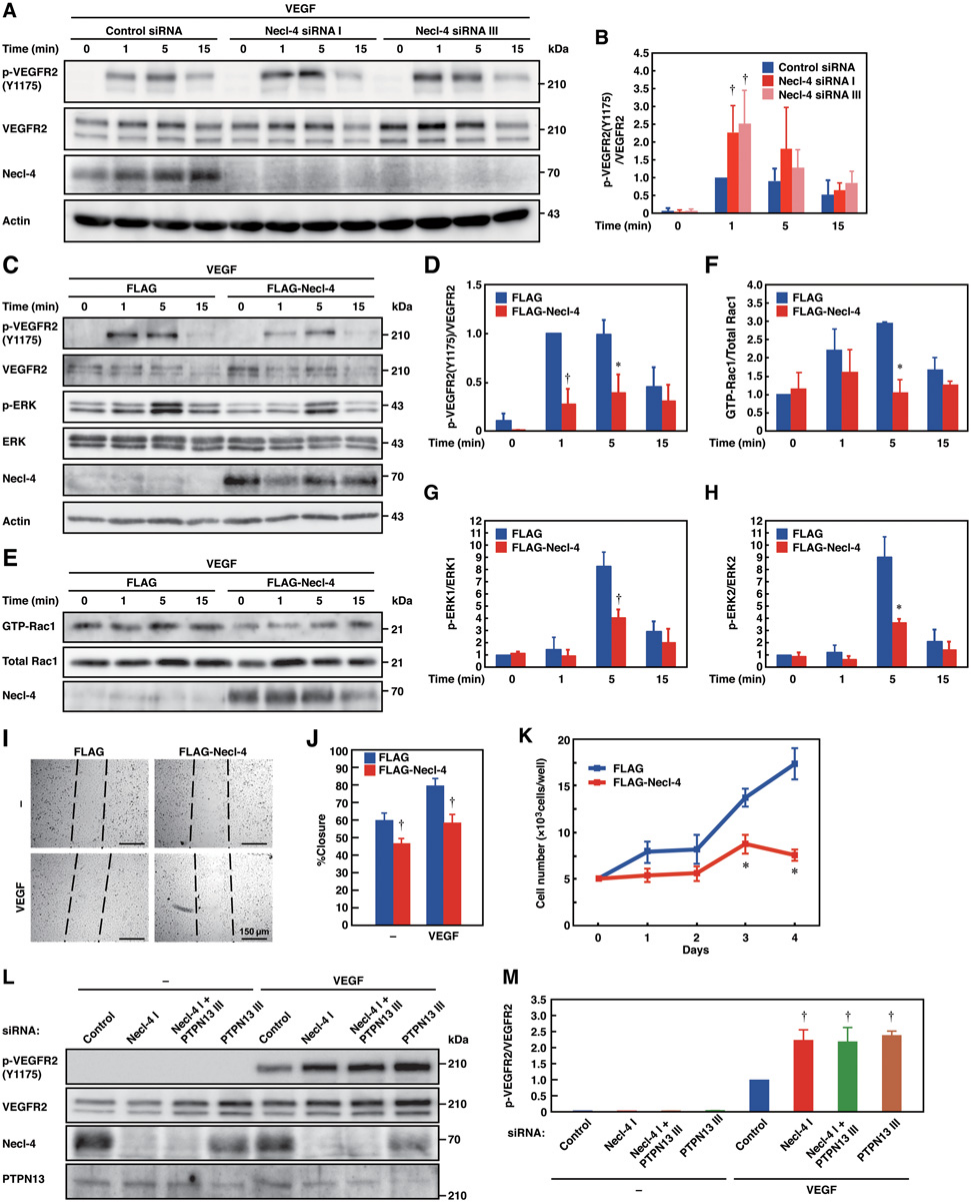
Necl-4 inhibits VEGFR2 activation, signaling, and cellular responses in confluently cultured ECs. **A and B**, Enhanced phosphorylation of VEGFR2 by Necl-4-knockdown. HUVECs transfected with control or Necl-4 siRNAs were cultured under confluent conditions in the presence or absence of 50 ng/ml VEGF for the indicated periods of time. Cell lysates were subjected to Western blotting using the indicated antibodies (*n* = 4). †*P*<0.01 vs. control siRNA. **C–H**, Reduced phosphorylation and signaling of VEGFR2 by Necl-4-overexpression. HUVECs transfected with FLAG or FLAG-Necl-4 were cultured under sparse conditions in the presence or absence of 50 ng/ml VEGF for the indicated periods of time. Cell lysates were subjected to Western blotting using the indicated antibodies or pull-down assays using GST-PAK-CRIB (*n* = 4). *P<0.05; †*P*<0.01 vs. FLAG. **I and J**, Reduced movement by Necl-4-overexpression. HUVECs transfected with FLAG or FLAG-Necl-4 were plated onto collagen-coated culture dishes and subjected to wound-healing assays in the presence or absence of 50 ng/ml VEGF (*n* = 4). †*P*<0.01 vs. FLAG. **K**, Reduced VEGF-induced proliferation by Necl-4-overexpression. HUVECs transfected with FLAG or FLAG-Necl-4 were cultured on 24-well plates coated with collagen in EBM-2 plus 2% FBS in the presence of 50 ng/ml VEGF. At the indicated time points, HUVECs were detached and the number of the cells was counted (*n* = 3). *P<0.05 vs. FLAG. **L and M**, Involvement of PTPN13 in the enhanced phosphorylation of VEGFR2 by Necl-4-knockdown. HUVECs transfected with control, Necl-4, PTPN13, or Necl-4 plus PTPN13 siRNAs were cultured under confluent conditions in the presence or absence of 50 ng/ml VEGF for 1 min and their lysates were subjected to Western blotting using the indicated antibodies (*n* = 3). †*P*<0.01 vs. control siRNA.

We then examined the role of the interaction of Necl-4 with VEGFR2 in VEGFR2 activation, signaling, and cellular responses by either knocking down or overexpressing Necl-4. Necl-4-knockdown (S3 Fig) enhanced under sparse conditions, whereas Necl-4-overexpression decreased VEGF-induced phosphorylation of VEGFR2 under confluent conditions (Fig 4A–4D). Upon binding to VEGFR2, VEGF induces the activation of the phosphatidylinositol 3-kinase—Rac pathway for movement [33,34] and the phospholipase Cγ–protein kinase C—Raf—mitogen-activated protein kinase kinase (MEK)1/2-ERK1/2 pathway for proliferation [35]. Therefore, the activation of Rac1 and ERK1/2 was analyzed. Necl-4-overexpression decreased the VEGF-induced activation of these signaling molecules (Fig 4C and 4E–4H), movement, and proliferation (Fig 4I–4K). These results indicate that Necl-4 *cis*-interacts with VEGFR2 and inhibits its activation, signaling, and cellular responses.

We next examined whether PTPN13 was involved in the inhibitory effect of Necl-4 on the phosphorylation of VEGFR2. PTPN13-knockdown (S4 Fig) enhanced the VEGF-induced phosphorylation of VEGFR2, but double knockdown of Necl-4 and PTPN13 did not further enhance this phosphorylation (Fig 4L and 4M). These results indicate that Necl-4 inhibits the VEGF-induced phosphorylation of VEGFR2 through PTPN13.

### Necl-4 strongly interacts with VEGFR2 with no effect on VEGFR2 activation and enhancement of VEGFR2 signaling and cellular responses in sparsely cultured ECs

**Fig 5:**
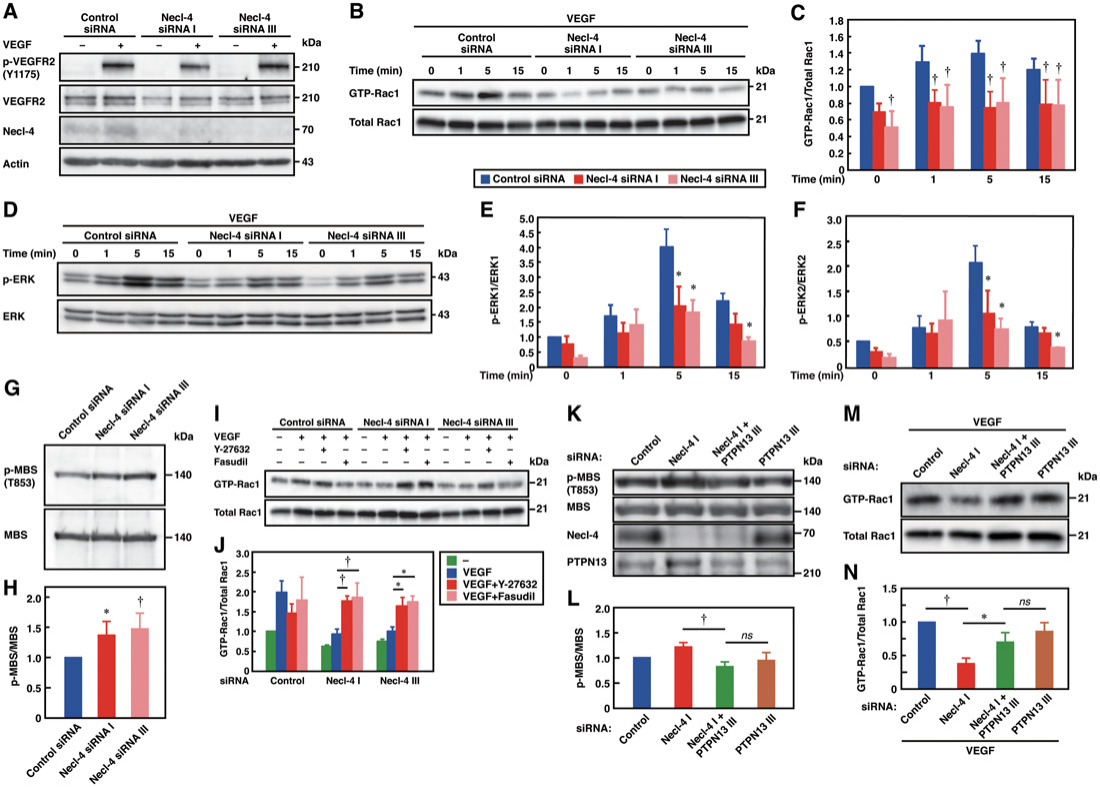
Necl-4 enhances the VEGFR2 signaling in sparsely cultured ECs. **A**, No effect of Necl-4-knockdown on the phosphorylation of VEGFR2 under sparse conditions. Lysates of HUVECs, transfected with control or Necl-4 siRNAs and cultured in the presence or absence of 50 ng/ml VEGF for 1 min, were subjected to Western blotting using the indicated antibodies. **B and C**, Reduced activation of Rac1 by Necl-4-knockdown. Lysates of HUVECs, transfected with control or Necl-4 siRNAs and cultured in the presence or absence of 50 ng/ml VEGF for the indicated periods of time, were subjected to pull-down assays using GST-PAK-CRIB (*n* = 4). †*P*<0.01 vs. control siRNA. **D–F**, Reduced phosphorylation of ERK by Necl-4-knockdown. Lysates of HUVECs, transfected with FLAG or FLAG-Necl-4 and cultured in the presence or absence of 50 ng/ml VEGF for the indicated periods of time, were subjected to Western blotting using the indicated antibodies (*n* = 4). *P<0.05; †*P*<0.01 vs. FLAG. **G and H**, Activation of ROCK by Necl-4-knockdown. Lysates of HUVECs transfected with control or Necl-4 siRNAs were subjected to Western blotting using the indicated antibodies. *P<0.05; †*P*<0.01 vs. control siRNA. **I and J**, Restoration of the reduced activation of Rac1 in Necl-4-knockdown HUVECs by ROCK inhibitors. Lysates of HUVECs, transfected with control or Necl-4 siRNAs, incubated with or without 10 μM Y-27632 or fasudil, and cultured in the presence or absence of 50 ng/ml VEGF for 5 min, were subjected to Western blotting using the indicated antibodies or pull-down assays using GST-PAK-CRIB (*n* = 3). *P<0.05; †*P*<0.01. **K and L**, Restoration of the activity of ROCK by additional knockdown of PTPN13. Lysates of HUVECs transfected with control, Necl-4, PTPN13, or Necl-4 plus PTPN13 siRNAs were subjected to Western blotting using the indicated antibodies (*n* = 3). †*P*<0.01; ns, not significant. **M and N**, Restoration of the activity of Rac1 by additional knockdown of PTPN13. Lysates of HUVECs, transfected with control, Necl-4, PTPN13, or Necl-4 plus PTPN13 siRNAs and cultured in presence of 50 ng/ml VEGF for 5 min, were subjected to pull-down assays using GST-PAK-CRIB (*n* = 3). *P<0.05; †*P*<0.01; ns, not significant.

Because Necl-4 was localized at the leading edges of sparsely cultured ECs, we examined whether Necl-4 coordinated VEGFR2 activation, signaling, and cellular responses under sparse conditions. Although Necl-4 strongly interacted with VEGFR2 (Fig 3B), Necl-4-knockdown did not influence the VEGF-induced phosphorylation of VEGFR2 (Fig 5A). Necl-4-knockdown decreased the basal and VEGF-stimulated activities of Rac1 (Fig 5B and 5C). Moreover, Necl-4-knockdown did not influence the basal phosphorylation levels of ERK1/2, but decreased the VEGF-induced increase in the phosphorylation of ERK1/2 (Fig 5D–5F). These results indicate that Necl-4 increases the basal activity of Rac1 and the VEGF-induced activation of Rac1 and ERK1/2 under sparse conditions.

Because there is a crosstalk between Rho-associated protein kinase (ROCK) and Rac1 whereby ROCK inhibits the activity of Rac1 [36], we examined whether ROCK was activated by Necl-4-knockdown. The phosphorylation of Thr^853^ (T853) residues of the MBS of myosin light chain phosphatase was increased by Necl-4-knockdown, indicating the activation of ROCK (Fig 5G and 5H). The decreased activation of Rac1 in Necl-4-knockdown cells was restored by specific ROCK inhibitors, Y-27632 and fasudil (Fig 5I and 5J). Consistent with this, Necl-4-knockdown increased formation of stress fibers and focal adhesions, which were regulated by ROCK [37,38], and the increased formation of stress fibers and focal adhesions was restored by Y-27632 and fasudil (S5 Fig). However, the decreased phosphorylation of ERK1/2 was not restored by Y-27632 or fasudil (data not shown). These results indicate that the increased activity of ROCK inhibits the activation of Rac1, but is not involved in the decreased activation of ERK1/2 by Necl-4-knockdown. In Necl-4-knockdown cells, additional knockdown of PTPN13 restored the increased phosphorylation of MBS (Fig 5K and 5L) and the decreased activation of Rac1 (Fig 5M and 5N). These results indicate that PTPN13 mediates the Necl-4-knockdown-induced activation of ROCK.

**Fig 6:**
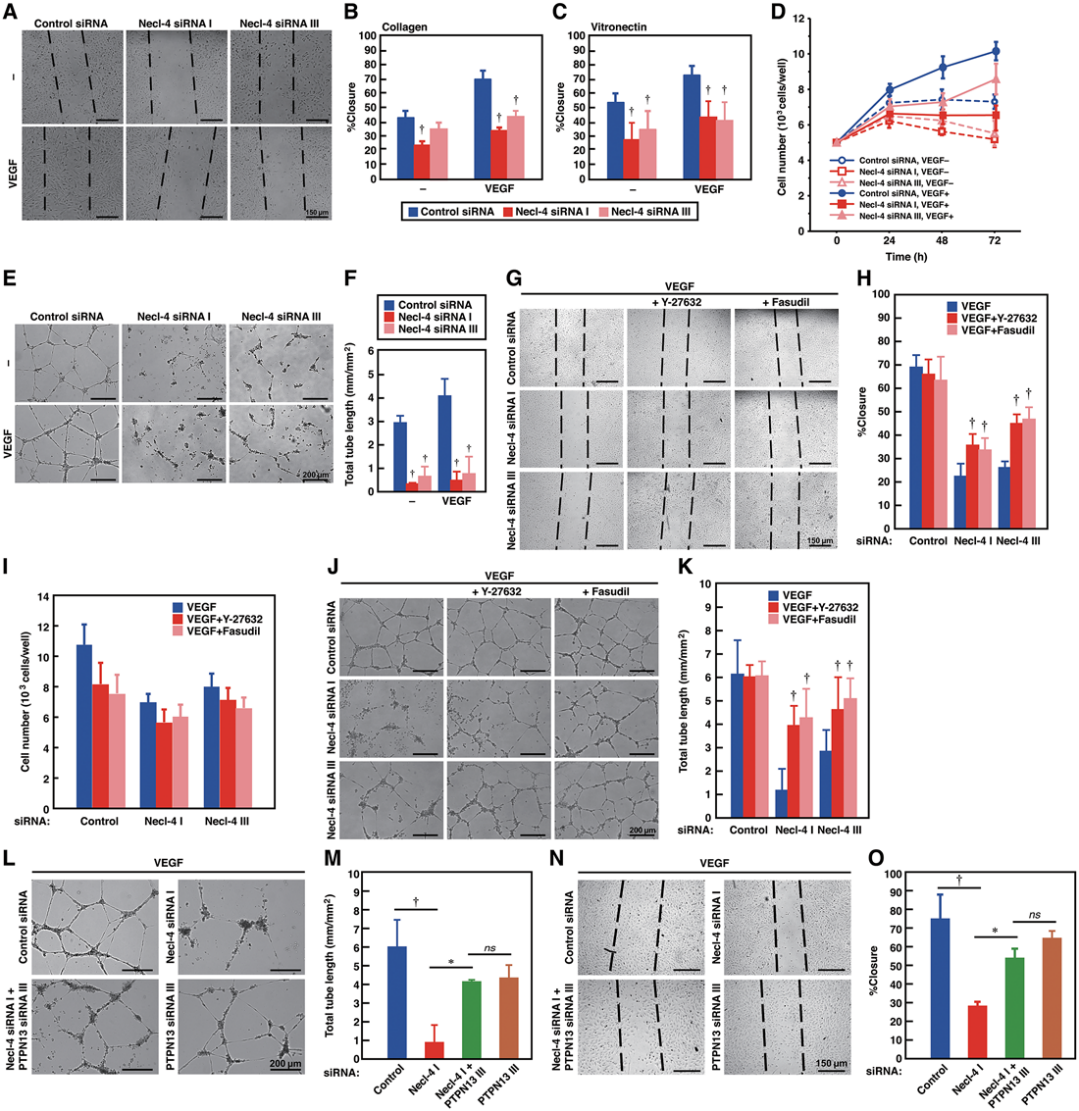
Necl-4 enhances cellular responses in sparsely cultured ECs. **A–C**, Reduced movement by Necl-4-knockdown. HUVECs transfected with control or Necl-4 siRNAs were subjected to wound-healing assays in the presence or absence of 50 ng/ml VEGF. Culture dishes were coated with collagen (**A and B**) or vitronectin (**C**) (*n* = 3). †*P*<0.01 vs. control siRNA. **D**, Reduced proliferation by Necl-4-knockdown. HUVECs transfected with control or Necl-4 siRNAs were cultured on 96-well plates coated with type I collagen in EBM-2 plus 2% FBS in the absence or presence of 50 ng/ml VEGF. At the indicated time points, the numbers of the cells were quantified by crystal violet staining (*n* = 3). (**E and F**, Reduced tubulogenesis by Necl-4-knockdown. HUVECs, transfected with control or Necl-4 siRNAs were subjected to Matrigel network formation assays in the presence or absence of 50 ng/ml VEGF (*n* = 4). †*P*<0.01 vs. control siRNA. **G, H, J, and K**, Restoration of the reduced movement and tubulogenesis of Necl-4-knockdown HUVECs by ROCK inhibitors. HUVECs, transfected with control or Necl-4 siRNAs and incubated with or without 10 μM Y-27632 or fasudil, were subjected to wound-healing assays (**G and H**) (*n* = 3) or Matrigel network formation assays (**J and K**) (*n* = 4) in the presence of 50 ng/ml VEGF. †*P*<0.01 vs. VEGF. **I**, No effects of ROCK inhibitors on the reduced proliferation of Necl-4-knockdown HUVECs. HUVECs, transfected with control or Necl-4 siRNAs and incubated with or without 10μM Y-27632 or fasudil, were cultured on 24-well plates coated with collagen in EBM-2 plus 2% FBS in the presence of 50 ng/ml VEGF. After 48 h, the numbers of the cells were quantified by crystal violet staining (*n* = 3). **L–O**, Restoration of the reduced tubulogenesis and movement of Necl-4-knockdown HUVECs by additional knockdown of PTPN13. HUVECs, transfected with control, Necl-4, PTPN13, or Necl-4 plus PTPN13 siRNAs, were subjected to Matrigel network formation assays (**L and M**) (*n* = 3) or wound-healing assays (**N and O**) (*n* = 3) in the presence of 50 ng/ml VEGF. *P<0.05; †*P*<0.01; ns, not significant.

We next investigated the role of Necl-4 in cellular responses under sparse conditions. In the presence or absence of VEGF, closure of scraped areas was delayed in Necl-4-knockdown ECs (Fig 6A–6C). Similarly, cell proliferation and tubulogenesis in the presence or absence of VEGF were significantly reduced in Necl-4-knockdown ECs (Fig 6D–6F). The reduced movement and tubulogenesis were partly restored by Y-27632 and fasudil (Fig 6G, 6H, 6J and 6K), whereas the reduced proliferation was not affected by these inhibitors (Fig 6I). These results indicate that the activation of ROCK is involved in the reduction of movement and tubulogenesis, but not of proliferation, in Necl-4-knockdown ECs. In the Necl-4-knockdown ECs, additional knockdown of PTPN13 partly restored the reduced movement and tubulogenesis (Fig 6L–6O), the formation of stress fibers, and the defective formation of membrane protrusions, such as lamellipodia and peripheral ruffles (S6 Fig). Taken together, these results indicate that although Necl-4 strongly interacts with VEGFR2, it does not influence its activation, but enhances its signaling and cellular responses though the PTPN13-ROCK pathway in sparsely cultured ECs.

## Discussion

We showed here for the first time cell density-dependent differential localization of Necl-4. Under sparse conditions, Necl-4 was localized at the leading edges of migrating cells. Because Necl-4 interacted with VEGFR2 is localized at the leading edges of ECs [23,24], this interaction may recruit Necl-4 to the leading edges. When two migrating ECs collide, they initiate cell—cell adhesion. Because ECs express nectin-2, nectin-3, Necl-4, Necl-5, and VE-cadherin (Fig 2A), various combinations of *trans*-interactions of these molecules, such as those between nectin-2 and nectin-2, between nectin-3 and nectin-3, between Necl-4 and Necl-4, between nectin-2 and nectin-3, and between nectin-3 and Necl-5, initiate cell—cell adhesion [8,9], and subsequently *trans*-interaction between VE-cadherin and VE-cadherin causes the formation of AJ. Thus, Necl-4 is localized at cell—cell contact sites as a CAM [16] and may help retain VEGFR2 at these sites. Similar to Necl-4, Necl-5 is localized at leading edges of moving cells [12,23], but unlike Necl-4, it disappears from the plasma membrane upon cell—cell adhesion. Similar to Necl-4, E-cadherin and VE-cadherin are localized at cell—cell junctions when cells are confluent but, unlike Necl-4, they are diffusely distributed on the plasma membrane and not accumulated at leading edges of moving cells. Thus, Necl-4 is localized in a novel way that is different from other CAMs that are implicated in contact inhibition.

We also showed here that the expression of Necl-4 was regulated in a cell density-dependent manner. The expression of Necl-4 varied reversibly as it was down- or up-regulated depending on cell density. Necl-4 was down-regulated in many human cancer cell lines, but this down-regulation was irreversible, likely by DNA methylation [14,16,18]. Because both Necl-4 mRNA and protein levels were increased by confluence, transcriptional regulation is likely involved in the up-regulation of Necl-4 protein. Our results suggest that the up-regulation of Necl-4 protein was dependent on Rap1 and afadin. Because cell—cell adhesion initiated by the *trans*-interactions of nectins is known to induce the activation of Rap1, which then binds to and activates afadin [39], it could be hypothesized that the nectin-mediated cell—cell adhesion initiates the signals leading to the up-regulation of Necl-4 protein. In the process of cell—cell adhesion formation, it was reported that in MDCK cells, Rac1 is transiently activated by nectins during the initiation of their *trans*-interactions and then inactivated once the *trans*-interactions are established [40]. Therefore, it is possible that Necl-4 may be involved in the inactivation of Rac1 after the *trans*-interactions of nectins are established. We also demonstrated here that Necl-5, nectin-2, and nectin-3 were down-regulated, whereas VE-cadherin remained unchanged by confluence. These results were in agreement with the earlier observations that upon cell—cell adhesion Necl-5 is down-regulated by clathrin-dependent endocytosis [13], *VE-cadherin* gene expression remains unchanged [41], and *nectin-2* and *nectin-3* gene expression is down-regulated [42]. Thus, the expression of Necl-4 is regulated in a novel way that is different from other CAMs implicated in contact inhibition.

We previously showed that Necl-4 inhibited the phosphorylation of ErbB3 and the ErbB2/ErbB3 signaling though PTPN13 [17]. Here we extended this observation and showed that Necl-4 inhibited the phosphorylation of VEGFR2 and its signaling though PTPN13. Similar to the action of the Necl-4 and PTPN13, VE-cadherin interacted with VEGFR2 and inhibited its phosphorylation through density-enhanced protein tyrosine phosphatase-1 (DEP1), a tyrosine phosphatase [43]. Thus, the Necl-4–PTPN13 and VE-cadherin—DEP1 signalings regulate the contact-dependent inhibition of the phosphorylation of VEGFR2. However, these mechanisms may work at different stages. Because the *trans*-interaction between Necl-4 is suggested to precede the *trans*-interaction between VE-cadherin upon cell—cell adhesion, the phosphorylation of VEGFR2 may be first inhibited by the Necl-4–PTPN13 signaling, resulting in reduced cell movement and proliferation, and further inhibited by the VE-cadherin—DEP1 signaling, resulting in the complete termination of cell movement and proliferation. Under confluent conditions, the VEGF-induced phosphorylation of VEGFR2 was inhibited despite the decreased interaction with Necl-4. This may be owing to the increased interaction between PTPN13 and VEGFR2. The reason why the interaction between PTPN13 and VEGFR2 was increased despite the down-regulation of PTPN13 under confluent conditions is currently unknown. However, the *trans*-interaction between Necl-4 might increase the interaction between Necl-4 and PTPN13, leading to the increased interaction between PTPN13 and VEGFR2. Another possibility is that the phosphatase activity of PTPN13 could increase under confluent conditions for some unknown reason.

In the present study, Necl-4 stimulated the basal and VEGF-induced cell movement through the PTPN13–ROCK—Rac1 pathway. This reveals a novel signaling pathway that regulates the activity of Rac1. The basal activities of ROCK and Rac1 may be responsible for the basal VEGF-independent cell movement. Compared with the activated state, not so much attention has been paid to the basal activity, but recent studies revealed its significance on various cellular functions. For example, in mouse olfactory sensory neurons, basal G-protein-coupled receptor activity determined the receptor-instructed axonal projection to olfactory bulb glomerulus [44]. In rat hippocampal neurons, the basal activity of PKA was essential for maintaining phosphorylation of neuronal L-type voltage-gated Ca^2+^ channels and their coupling to the calcineurin-nuclear factor of activated T-cell (NFAT) pathway [45]. How Necl-4 regulates the activity of ROCK is unknown, but Necl-4 might inhibit Rho guanine nucleotide exchange factors (GEFs) and/or stimulate Rho GTPase-activating proteins (GAPs) through PTPN13, thereby inhibiting the activity of Rho, or directly inhibit the tyrosine-phosphorylation of ROCK, thereby increasing its binding to RhoA [46]. Meanwhile, Necl-4 enhanced the VEGF-induced, but not the basal, activation of ERK1/2; PTPN13 and ROCK were not involved in this enhancing mechanism (data not shown). Because the VEGF-induced activation of ERK1/2 requires receptor endocytosis [47,48], Necl-4 might regulate the endocytosis of VEGFR2 through protein 4.1, which can interact with Necl-4 [18] and regulate receptor endocytosis [49,50].

Similar to Necl-4, PTPN13 has two different roles in cell movement depending on its localization. When PTPN13 was localized at leading edges, PTPN13 inhibited cell movement, which is consistent with the earlier observation that PTPN13 inhibited lysophosphatidic acid-induced cell movement [51]. In contrast, when PTPN13 was localized at cell—cell contact sites, PTPN13 may dephosphorylate receptor tyrosine kinases or tyrosine-phosphorylated adaptor proteins, thereby turning off their signals to inhibit cell movement. PTPN13 interacted with the cytoplasmic tail of Necl-2, similar to the interaction with Necl-4, and thereby inhibited the heregulin-induced ErbB3/ErbB2 signaling and ErbB4 activity [52,53]. The similar localization to PTPN13 has been seen in other CAM-associated molecules, such as afadin [29], Rap1 [54], and ZO-1 [55], but in contrast to PTPN13 they enhanced cell movement when accumulated at leading edges. These inhibitory effects of PTPN13 on cell movement may account for its tumor-suppressive role.

**Fig 7:**
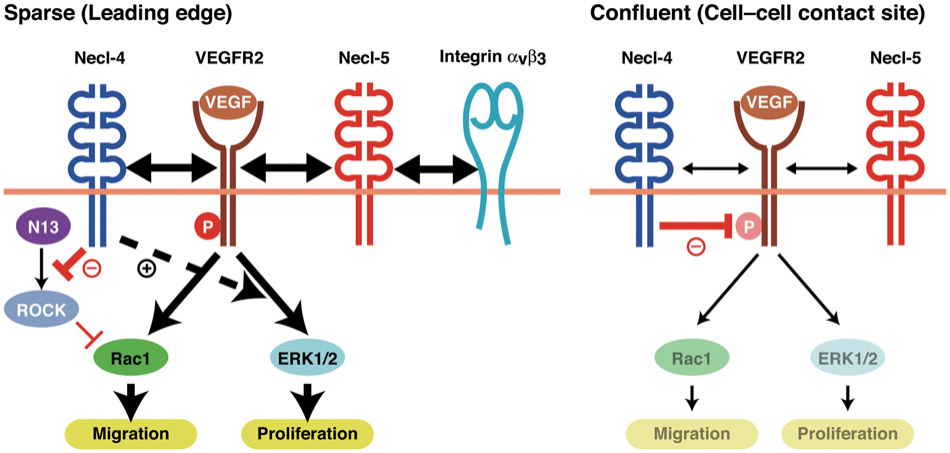
Model of the regulation of the phosphorylation and signaling of VEGFR2 by Necl-4 and PTPN13. The details are described in the Discussion. N13, PTPN13; P, phosphorylation.

We presented a novel mechanism by which Necl-4 coordinates cell movement, cell proliferation, and contact inhibition. A schematic model for the mode of actions of Necl-4 on the VEGF-induced signaling in comparison with those previously described for Necl-5 [23] is shown (Fig 7). When cells are freely moving and proliferating without adhesion, Necl-4 and Necl-5 are localized at the leading edges to enhance movement and proliferation by distinct mechanisms. Necl-5 enhances the *cis*-interaction between VEGFR2 and integrin α_V_β_3_, thereby augmenting the VEGF-induced phosphatidylinositol 3-kinase pathway and leading to increased cell movement [23]. Meanwhile, Necl-4 likely inhibits the PTPN13-induced activation of ROCK, thereby facilitating the VEGF-induced activation of Rac1 and leading to increased cell movement; concurrently Necl-4 enhances the VEGF-induced activation of ERK1/2, leading to increased cell proliferation, although Necl-5 regulates proliferation in an ERK-independent manner [23]. When cells adhere to each other, Necl-5 is down-regulated, which causes the loss of enhancement of the signaling pathways for movement and proliferation, and Necl-4 is subsequently up-regulated and localized at cell—cell contact sites, where it causes the inactivation of these signaling pathways by inhibiting the phosphorylation of VEGFR2 through PTPN13.

On the basis of our findings, we propose the idea for staging of contact inhibition (S7 Fig). Contact inhibition is initiated by a loss of Necl-5-dependent enhancement of the signaling pathways for movement and proliferation [13]. Then, these signaling pathways are inactivated by Necl-4. At the maintenance stage, cadherins play a role: because the binding affinity of the homophilic *trans*-interaction between cadherins is much stronger than that of the heterophilic *trans*-interaction between nectins [12,56], the homophilic *trans*-interaction of cadherins expressed in neighbouring cells physically terminates cell movement, and simultaneously blocks the signaling for proliferation, leading to the cessation of proliferation [43,57]. Once TJs and apico-basal cell polarity are established, contact inhibition is further maintained by apical polarity complex-associated merlin and angiomotin, a member of the motin family; these molecules regulate the Hippo pathway [58]. At the initiation stage, the affinity for cell—cell adhesion is relatively weak and therefore cells can be reversibly detached. By contrast, at the maintenance stage where AJs and TJs are established, cell—cell adhesion is not easily detached. Thus, the present study revealed that Necl-4 could be a first key molecule that is involved in the late phase of the initiation stage. In addition, at the sequential stages of contact inhibition, the mode of action of the contact inhibition-related molecules on cell movement and proliferation, including Necl-5, cadherins, merlin, and the Hippo-YAP pathway, is essentially unidirectional. By contrast, Necl-4 showed bidirectional action, stimulation and inhibition of cell movement and proliferation. The present findings might be applicable to cell types other than ECs because Necl-4 was similarly up-regulated by confluence in epithelial cells in which Necl-4 negatively regulated the heregulin-induced ErbB2/ErbB3 signalling [17].

## Supporting Information

S1 Fig No cross-reaction among the anti-Necl antibodies. Lysates of HEK293 cells transfected with each Necl-expressing plasmid or untransfected were subjected to Western blotting using the indicated antibodies.
(EPS)

S2 Fig Involvement of Rap1 and afadin in the up-regulation of Necl-4 by confluence. HUVECs were transfected with indicated siRNAs and cultured under sparse (S, 25% confluence) or confluent (C, 100% confluence) conditions. Cell lysates were subjected to Western blotting using the indicated antibodies (**A**). RNAs extracted from HUVECs were subjected to qPCR (*n* = 4) (**B**). *P<0.05 vs. 25% confluence.
(EPS)

S3 Fig Knockdown of Necl-4 by siRNAs. Lysates of HUVECs transfected with control or Necl-4 siRNAs were subjected to Western blotting using the indicated antibodies (*n* = 3). †*P*<0.01 vs. un-transfected (-).
(EPS)

S4 Fig Knockdown of PTPN13 by siRNAs. Lysates of HUVECs transfected with control or PTPN13 siRNAs were subjected to Western blotting using the indicated antibodies (*n* = 3). *P<0.05 vs. un-transfected (-).
(EPS)

S5 Fig Increased formation of stress fibers and focal adhesions in Necl-4-knockdown ECs. A, Increased formation of stress fibres by Necl-4-knockdown. HUVECs, transfected with control or Necl-4 siRNAs and incubated with or without 10 μM Y-27632 or fasudil, were stained with phalloidin and DAPI. Representative images are shown (*n* = 3). **B**, Increased formation of focal adhesions by Necl-4-knockdown. HUVECs, transfected with control or Necl-4 siRNAs and incubated with or without 10 μM Y-27632 or fasudil, were stained with anti-vinculin mAb, phalloidin, and DAPI. Representative images are shown (*n* = 3).
(EPS)

S6 Fig PTPN13 is involved in the increased formation of stress fibers and the reduced formation of protrusions in Necl-4-knockdown ECs. **A**, Restoration of the increased formation of stress fibers in Necl-4-knockdown HUVECs by additional knockdown of PTPN13. HUVECs, transfected with control, Necl-4, PTPN13, or Necl-4 plus PTPN13 siRNAs, were stained with phalloidin and DAPI. Representative images are shown (*n* = 3). **B**, Restoration of the reduced formation of protrusions in Necl-4-knockdown HUVECs by additional knockdown of PTPN13. HUVECs, transfected with control, Necl-4, or Necl-4 plus PTPN13 siRNAs, were subjected to wound-healing assays in the presence of 50 ng/ml VEGF. Eight hours after wound-healing, HUVECs were stained with phalloidin. Representative low (upper lane) and high (lower lane) magnification images are shown (*n* = 3).
(EPS)

S7 Fig Sequential stages of contact inhibition. **A**, The initiation stage. Necl-5 enhances growth factor and integrin α_V_β_3_ complex-induced migratory and proliferative signalling. Contact inhibition is initiated by the loss of the enhancement of this migratory and proliferative signalling due to the endocytosis of Necl-5 by the *trans*-interaction between Necl-5 and nectin-3, which is followed by the inactivation of the migratory and proliferative signalling by inhibiting of growth factor receptor phosphorylation by Necl-4. **B**, The maintenance stage. After the establishment of AJs, contact inhibition is maintained by cadherins and catenins in cooperation with merlin and the Hippo pathway, and is further maintained by apical polarity complex-associated angiomotin and merlin after the establishment of tight junctions and apico-basal cell polarity.
(EPS)
